# Endoscopic hematoma evacuation for acute subdural hematoma in a young patient: a case report

**DOI:** 10.1002/ams2.295

**Published:** 2017-07-17

**Authors:** Atsushi Kuge, Daisuke Tsuchiya, Shigeki Watanabe, Mitsuya Sato, Toshihiko Kinjo

**Affiliations:** ^1^ Department of Emergency Medicine Okitama Public General Hospital Yamagata Japan; ^2^ Department of Neurosurgery Okitama Public General Hospital Yamagata Japan

**Keywords:** acute subdural hematoma, endoscope, head trauma, minimally invasive surgery, young patient

## Abstract

**Case:**

The standard treatment for acute subdural hematoma (ASDH) is large craniotomy; decompressive craniectomy may also be carried out, if needed, to prevent secondary brain damage. Recently, an endoscopic procedure for elderly patients with ASDH was carried out and reported; its safety and effectiveness were emphasized because of minimal invasiveness. We report a young case and discuss its difficulties and tips.

A 31‐year‐old man was found to be in a state of general convulsion. At the time of admission, we observed severe consciousness disturbance, anisocoria, and left hemiparesis. Computed tomography showed a massive subdural hematoma with marked midline shift.

**Outcome:**

Osmotherapy and emergency trepanation improved anisocoria. An endoscopic procedure under local anesthesia was sequentially selected. After surgery, the patient's symptoms clearly improved.

**Conclusion:**

Although the standard treatment for ASDH is craniotomy, endoscopic surgery may be useful in some cases.

## Introduction

The standard treatment for acute subdural hematoma (ASDH) is large craniotomy and decompressive craniectomy to prevent secondary brain damage. Recently, an endoscopic procedure has been applied for elderly patients because of its minimal invasiveness.^1^, ^2^, ^3^, ^4^ Although there have been no previous reports of this endoscopic procedure being applied to young patients, we report a case and discuss its difficulties and tips to solve these problems.

## Case

**Figure 1:**
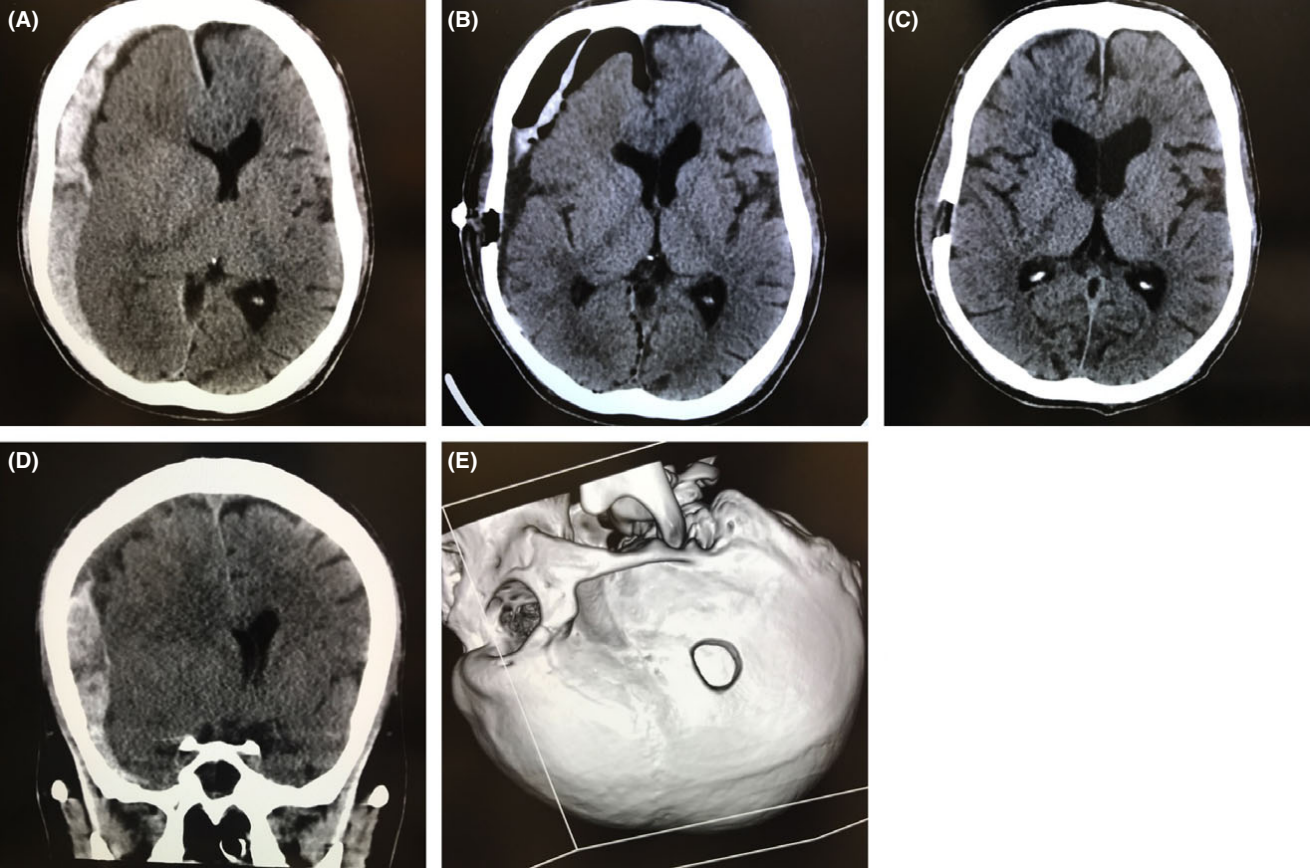
Preoperative and postoperative computed tomography scans in a 31‐year‐old man with acute subdural hematoma, with a history of schizophrenia and epilepsy. A, D, Massive hematoma observed preoperatively. B, C, One day (B) and 1 month (C) following endoscopic surgery, the mass effect was decreased. E, Surgical window for endoscopic procedure, showing the small hole (diameter, 20 mm) for inserting and maneuvering endoscopic instruments.

A 31‐year‐old man with a history of schizophrenia and epilepsy was found in a state of general convulsion. He was transferred to our hospital by emergency medical service. At the time of admission, his Glasgow Coma Scale score was 6 (E1V1M4). Neurologically, he showed anisocoria (right 5 mm, left 3 mm), bilateral slow light reflex, and left hemiplegia. His respiratory pattern was irregular. After oral intubation, computed tomography (CT) was carried out. The CT scan showed a massive subdural hematoma on the right with marked midline shift (Fig. 1). There was no swirl sign, massive brain contusion, or intracerebral hematoma. Laboratory data were: platelets, 11 × 10^3^/μL; prothrombin time – international normalized ratio, 1.15; activated partial thromboplastin time, 31.7 s; D‐dimer, 3 μg/mL. He had no hemorrhagic diathesis.

Mannitol infusion and emergency trepanation in the emergency room made slight improvements in consciousness disturbance (Glasgow Coma Scale E1VTM5) and anisocoria disappeared. Although craniotomy with general anesthesia was requested, this was not possible because many operations were being carried out at that time with general anesthesia. Therefore, an endoscopic procedure under local anesthesia was selected. We started the endoscopic surgery 1 h after trepanation without aggravation of neurological symptoms. The patient was sedated with dexmedetomidine and fentanyl. If the endoscopic procedure had proved impossible because of uncontrolled bleeding and acute brain swelling, simultaneously craniotomy was prepared.

The procedure was undertaken in the emergency room. The patient was placed in the supine position with his head rotated to the left, and a small key hole (diameter, 20 mm) was made (Fig. 1E). This small hole was sufficient for inserting and maneuvering endoscopic instruments.

**Figure 2:**
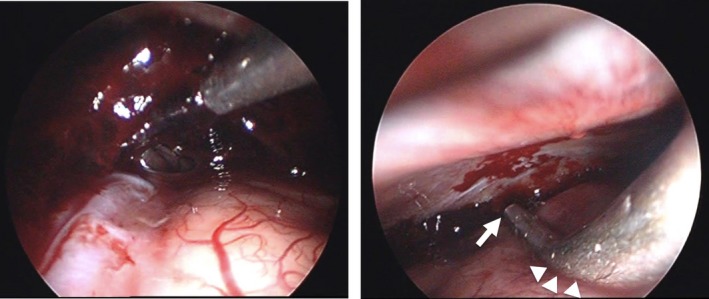
Intraoperative photographs of endoscopic hematoma evacuation for acute subdural hematoma in a 31‐year‐old man. Left image, thick dark red clot located between the dura and brain surface. Right image, a malleable suction cannula retracts the brain surface in order to secure surgical space (arrow) and the tip of the cannula was turned toward the dura to prevent brain damage from surgical procedures (arrowheads).

A rigid scope with a tip angle of 0 or 30° (Olympus, Tokyo, Japan) clearly showed a residual dark red clot (Fig. 2, left). The clot was evacuated using a suction tube and was irrigated with artificial cerebrospinal fluid (Otsuka Pharmaceutical, Tokushima, Japan). Although an active bleeding point could not be seen, almost all of the clot was removed and complete hemostasis was achieved. For hemostasis against the bleeding point, we prepared flexible suction monopolar which was insulated and TAKE‐APART® bipolar forceps (Karl Storz Endoskope, Tuttlingen, Germany). Acute brain swelling did not develop. The dura mater was closed loosely and covered with Surgicel (Johnson & Johnson, New Brunswick, NJ, USA). A drainage tube was not inserted. The wound was closed with Precise Vista Lite (3M, Tochigi, Japan).

Postoperative CT revealed almost total removal of hematoma and improved mass effect (Fig. 1B). The patient's consciousness disturbance and left hemiplegia improved immediately. He stayed 8 days in our hospital and was discharged without neurological deficit.

## Discussion

Acute subdural hematoma is well known as a severe prognostic factor of head trauma that requires a large craniotomy. Adverse effects such as a blood loss and brain swelling could occur.^5^, ^6^ Although the usefulness of endoscopic surgery as a minimally invasive procedure for subdural hematoma has been reported in elderly patients,^1^, ^2^, ^3^, ^4^ there has been no report of endoscopic surgery applied for ASDH in young patients.

As an indication of endoscopic surgery for SDH, Karakhan *et al*. reported the contraindications of endoscopic surgery as: (i) widespread brain laceration; (ii) large bleeding vessels; (iii) brain prolapse; (iv) calcification of hematoma.^7^ Yokosuka *et al*. reported the indications of endoscopic surgery as: (i) the presence of symptoms; (ii) age older than 70 years; (iii) absence of moderate or massive brain contusion/hematoma; (iv) absence of an enlarging SDH; (v) no high risk of bleeding.^4^ Our case followed the indications for elderly patients as above, except for the factor of age.

It is important to secure a subdural space in order to carry out endoscopic procedures safely. Elderly patients have enough subdural space as a result of brain atrophy. This allows for safe access to the hematoma using an endoscope. Because our patient had been schizophrenic for a long time and had mild brain atrophy, there was enough surgical space to safely undertake the procedure.

The development of useful equipment is important. Yokosuka created an irrigation suction cannula with a malleable nozzle to improve procedures in the subdural space.^4^ We also used malleable suction in order to retract the brain surface using a curved section of a suction cannula. This technique is very effective to evacuate the hematoma and manage hemostasis with insertion of some surgical instruments, even if mild brain swelling has occurred (Fig. 2, right).

Fortunately, our case had no remarkable brain swelling or massive bleeding during surgery, we were able to accomplish the endoscopic procedure with no serious difficulties.

We think that sufficient surgical space is one of the most important points to consider when performing endoscopic surgery for a young patient. It is crucial to review the preoperative images and other laboratory findings thoroughly (state of subdural space, obvious bleeding point, bleeding tendency, etc.).

## Conclusions

We report a case of ASDH treated using an endoscopic procedure. Although the standard treatment for ASDH is craniotomy, endoscopic surgery may be useful in some cases. Further experiences are needed for safe endoscopic surgery for young patients as a minimally invasive treatment.

## Disclosure

Conflict of Interest: None declared. The patient received an explanation of this article, and gave informed consent.
